# The role of oxygen transport in atherosclerosis and vascular disease

**DOI:** 10.1098/rsif.2019.0732

**Published:** 2020-04-01

**Authors:** John Tarbell, Marwa Mahmoud, Andrea Corti, Luis Cardoso, Colin Caro

**Affiliations:** 1 Biomedical Engineering Department, The City College of New York, New York, NY, USA; 2 Department of Bioengineering, Imperial College London, London, UK

**Keywords:** hypoxia, atherosclerosis, vascular stent, helical stent, vasa vasorum, oxygen transport

## Abstract

Atherosclerosis and vascular disease of larger arteries are often associated with hypoxia within the layers of the vascular wall. In this review, we begin with a brief overview of the molecular changes in vascular cells associated with hypoxia and then emphasize the transport mechanisms that bring oxygen to cells within the vascular wall. We focus on fluid mechanical factors that control oxygen transport from lumenal blood flow to the intima and inner media layers of the artery, and solid mechanical factors that influence oxygen transport to the adventitia and outer media via the wall's microvascular system—the vasa vasorum (VV). Many cardiovascular risk factors are associated with VV compression that reduces VV perfusion and oxygenation. Dysfunctional VV neovascularization in response to hypoxia contributes to plaque inflammation and growth. Disturbed blood flow in vascular bifurcations and curvatures leads to reduced oxygen transport from blood to the inner layers of the wall and contributes to the development of atherosclerotic plaques in these regions. Recent studies have shown that hypoxia-inducible factor-1α (HIF-1α), a critical transcription factor associated with hypoxia, is also activated in disturbed flow by a mechanism that is independent of hypoxia. A final section of the review emphasizes hypoxia in vascular stenting that is used to enlarge vessels occluded by plaques. Stenting can compress the VV leading to hypoxia and associated intimal hyperplasia. To enhance oxygen transport during stenting, new stent designs with helical centrelines have been developed to increase blood phase oxygen transport rates and reduce intimal hyperplasia. Further study of the mechanisms controlling hypoxia in the artery wall may contribute to the development of therapeutic strategies for vascular diseases.

## 1. Introduction and background

The hypoxia theory of atherosclerosis proposes that an imbalance between the demand for and supply of oxygen in the arterial wall is a key factor in the development of intimal hyperplasia and atherosclerotic plaques. Recent review papers [1–3] have described the biomolecular mechanisms that advance atherosclerotic plaques in the presence of hypoxia. There is substantial evidence that there are regions within the atherosclerotic plaque in which significant hypoxia exists that may change the function, metabolism and responses of many of the cell types found within the developing plaque, and dictate whether the plaque will evolve into a stable or unstable phenotype.

**Figure 1.**
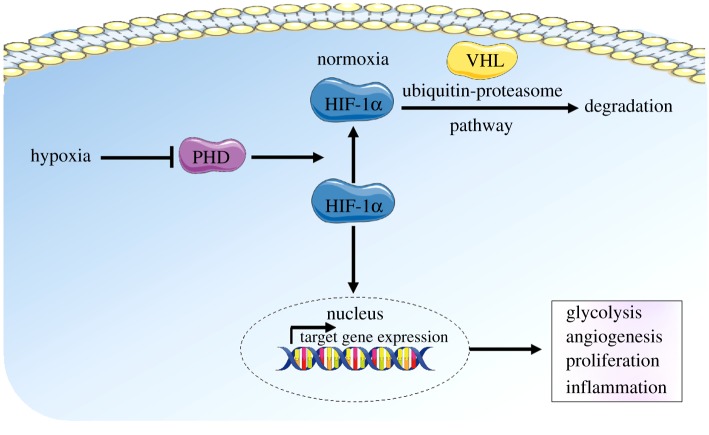
Schematic of hypoxic signalling. In normoxia, HIF-1α becomes targeted for degradation through proline hydroxylation by HIF prolyl hydroxylase (PHD). This results in a conformational change in HIF-1α, promoting its binding to VHL E3 ligase complex, and targeting HIF-1α for ubiquitination and rapid proteasomal degradation. In hypoxia, HIF-1α degradation is inhibited, resulting in its accumulation in the cells, and leading to its nuclear translocation and HIF1-α target gene expression. HIF-1α target genes mediate changes in cell function, including proliferation, angiogenesis, glycolysis and inflammation.

Hypoxia-inducible factor-1α (HIF-1α), is a transcription factor that orchestrates the hypoxic response in cells and has been regarded as the ‘master regulator of hypoxia’ (figure 1). Under normoxic conditions (physiological levels of oxygen—greater than about 5% or 40 mm Hg), HIF-1α becomes targeted for degradation through proline hydroxylation by HIF prolyl hydroxylase (PHD) which results in a conformational change promoting its binding to Von Hippel–Lindau disease tumour suppressor protein (VHL) E3 ligase complex, which in turn targets HIF-1α for ubiquitination and rapid proteasomal degradation. In hypoxia, HIF-1α degradation is inhibited, resulting in its accumulation in the cells, leading to its nuclear translocation and HIF-1α target gene expression. HIF-1α target genes mediate inflammation, proliferation, angiogenesis and glycolysis [3].

Inflammatory disease states are often characterized as either being a result of tissue hypoxia or in activating hypoxia [4]. Thus, it is not surprising that hypoxia and inflammation share common signalling pathways; of major importance is NF-кB activation. It was first reported in 1994 by Koong *et al.* [5] that hypoxia activates NF-кB signalling by triggering the degradation of inhibitory IкB-a, resulting in the release of p65 (RelA) from the inhibitory complex and translocation into the nucleus where it promotes the transcription of NF-кB target genes. Since then, there have been numerous studies that demonstrate the activation of the inflammatory NF-кB pathway in hypoxia (reviewed in [4,6]). Consistent with this, it has also been shown that NF-кB signalling triggers HIF-1α activation in immune cells. In response to macrophage stimulation by bacterial infection, lipopolysaccharides (LPS) or hypoxia, active NF-кB signalling triggers the activation of HIF-1α [7,8]. In endothelial cells, non-canonical hypoxic signalling triggered by disturbed blood flow in the vasculature results in the activation of HIF-1α through NF-кB [9]. Interestingly, both NF-кB and HIF-1α can be activated by the same stimuli, this includes proinflammatory cytokines such as TNF-α and interleukin-6, oxidative stress and disturbed blood flow [8,9].

In concert with inflammation, hypoxia also triggers glucose metabolic changes in cells. Under low oxygen levels this change in metabolism is required to maintain adequate ATP production in cells [10]. Under inflammatory conditions, HIF-1α triggers the activation of glycolysis genes in endothelial cells [9]. In macrophages, the production of LPS by bacterial infection triggers glycolysis through HIF-1α [7].

In endothelial cells, the glycolysis shift caused by HIF-1α gives rise to enhanced cell proliferation and inflammation [9,11]. HIF-1α also triggers endothelial–mesenchymal transition [12,13], a process that results in further enhancement of inflammation, proliferation and permeability and has been shown to trigger atherosclerosis [14,15]. All of these changes in endothelial cell function are a hallmark of a dysfunctional endothelium which leads to the development of atherosclerosis.

While the biomolecular mechanisms relating hypoxia to atherosclerosis have been well described, the biophysical mechanisms responsible for hypoxia and its localization to regions of the vasculature where atherosclerosis develops have received less attention. Thus, a major aim of this review is to elucidate the biophysical mechanisms responsible for hypoxia, and to suggest methods to ameliorate hypoxia that derive from biophysical understanding.

We begin with a discussion of the pathways for oxygen transport to the arterial wall emphasizing transport to the inner layers from luminal blood flow and the outer layers from the supporting microvascular network—the vasa vasorum (VV). The role of VV compression leading to medial layer hypoxia in vascular disease is elucidated and the pathways for inflammatory response and plaque development provided by the VV are described. Impaired blood phase oxygen transport characteristics in regions of branching and curvature where atherosclerotic plaques localize are then discussed. It is well known that these are regions of disturbed flow that induce endothelial cell dysfunction, and recent studies show that even HIF-1α is upregulated by disturbed flow. But, here, it is emphasized that these are typically regions of vessel wall hypoxia as well. The final sections of the review deal with vascular stenting that reduces downstream hypoxia but can induce vessel wall hypoxia, intimal hyperplasia and restenosis within the stented region. The effects of stent expansion on VV compression and reduced blood flow to the outer layers of the wall are reviewed. A final section describes the biophysical effects of a stent with a helical centreline that promotes enhanced oxygen transport to the inner layers of the wall by virtue of the secondary flows induced by the helical geometry and reduces intimal hyperplasia.

## 2. Pathways for oxygen transport to the arterial wall

**Figure 2.**
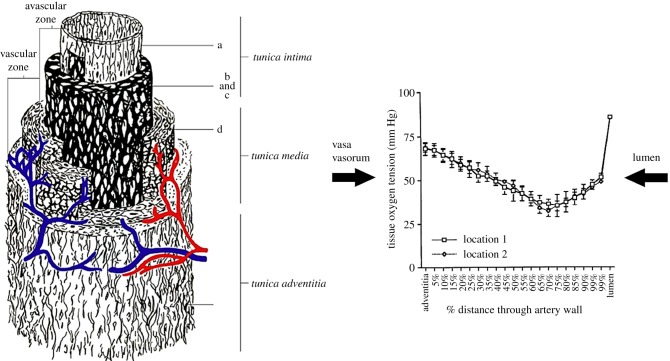
Two main pathways for oxygen transport to the blood vessel wall. (*a*) The intima and inner media are supplied from the luminal blood whereas the adventitia and outer media are supplied by the vasa vasorum [16]. (*b*) Arterial wall oxygen tension profile in the common carotid artery of a dog [17].

There are two principal pathways for oxygen transport to the arterial wall: the inner layers (intima and inner media) receive oxygen primarily from lumenal blood flow and the outer layers (adventitia and outer media) from the VV, a microvascular network whose principal function is to service the outer regions of thicker blood vessels (figure 2*a*).

Depending on their origin, VV are divided into three categories [18]: vasa vasorum interna (VVI), when the origin is the main lumen itself and the branching network remains inside the arterial wall; vasa vasorum externa (VVE), when the origin is located outside the vascular wall and comes from a major branch emerging from the main lumen; venous vasa vasorum (VVV), when the tree structure starts from a neighbouring vein. The pattern of VV penetration into the outer layers of the wall is dependent on the wall thickness. Wolinsky & Glagov [19] examined thoracic aortic segments of 12 mammalian species and observed that mammals whose aortas had 29 or fewer medial lamellar units had no demonstrable intramural vascular channels (penetrating VV); those whose aortas had more than 29 medial lamellar units, had medial VV [19]. Aortas with medial VV always had a subintimal medial zone devoid of VV. Remarkably, the thickness of the avascular zone was about 0.47 mm in all species, suggesting that diffusive transport from the inner most VV and the lumen is limited to this distance. Williams *et al*. [20] pointed out that in normal arteries, VV from the adventitia grow into the media of large arteries and veins and actively regulate blood flow to the wall of these vessels [20]. But in atherosclerotic arteries, VV proliferate into the intima-media, where they provide nutrition to the thickened artery. These neovascular channels are thin-walled, however, and may contribute to intraplaque haemorrhage, plaque disruption and mural thrombosis. Choi *et al*. [21] identified macrophages and microchannels in mild coronary atherosclerosis supporting the role of inflammation and VV proliferation in the early stage of coronary atherosclerosis.

Niinikoski *et al*. [22] demonstrated that a transarterial wall oxygen gradient is present in normal rabbit aortas with oxygen tensions falling from the adventitia, reaching a nadir at the junction of the inner one-third and outer two-thirds of the vessel wall. This was demonstrated more clearly with oxygen microelectrode measurements by Santilli *et al*. [17]. A typical oxygen tension profile across a healthy artery (in this case, the common carotid artery of a dog) is displayed in figure 2*b*. Oxygen tension drops precipitously from the lumen to the inner most regions of the wall, suggesting a significant oxygen transport resistance in the near wall region of the lumen. This is followed by a more gradual drop in oxygen tension in the inner layers as oxygen supplied from the lumen is consumed by the cells in the media (primarily smooth muscle cells in the un-diseased artery). The oxygen tension also drops gradually in the outer layers as fibroblasts and smooth muscle cells consume oxygen. The steadily falling oxygen tensions away from the adventitial surface suggest that the carotid artery wall is not supplied by any penetrating vessels from the VV. This is expected because the reported thickness of the vessel segment (0.204 mm) is well below the expected avascular zone thickness (0.47 mm) reported by Wolinsky & Glagov [19]. This was supported by light-microscopic examination which revealed VV only on the adventitial surface.

## 3. The role of oxygen transport pathways in vascular disease

### 3.1. Transport from the vasa vasorum

Early studies in the aortic media of dogs [23] indicated that VV provide a considerable amount of blood flow to the outer wall layers of the thoracic aorta. The vessels were responsive to physiologic stimuli as they dilated during infusion of adenosine and constricted during acute hypertension. They speculated that reduction of blood flow to the aortic wall via the VV in hypertension might contribute to aortic medial necrosis.

More recent studies in humans showed that aortic dissections initially developed in the outer third of the media, alongside VV, which showed sclerotic changes. It was suggested that dysfunction of VV in hypertension may play a key role in ischaemia and associated malnutrition of the aortic media leading to the initiation of dissection [24]. These observations are consistent with the earlier review of Baikoussis *et al*. [25].

Booth *et al*. [26] developed a model of atherosclerosis by positioning a hollow silastic collar around the carotid artery of cholesterol-fed rabbits resulting in macrophage and smooth muscle cell infiltration into the arterial subendothelium, foam cell formation and deposition of extracellular lipid, all in the presence of a morphologically intact endothelium. They proposed that the changes induced by the collar were mediated by obstruction of the adventitial VV and the creation of localized wall hypoxia. This concept of VV occlusion leading to outer wall ischaemia and progression to atherosclerosis was further elaborated by Martin *et al*. [27].

Analytical and numerical modelling of the deformation of venous and arterial VV were developed by Maurice *et al*. [28]. A nonlinear elastic vessel wall model showed that a normal range of intraluminal pressure induces a small deformation in the VV in arteries but a larger deformation in VVV. Increased luminal pressure was predicted to compress VV and reduce flow, thereby resulting in reduced oxygen transport to the outer wall. More recently, Ritman & Lerman [18] stressed that acute modulation of VV patency due to surrounding compressive forces within the vessel wall and due to variable tone in the smooth muscle affect the progression of atherosclerotic plaques.

Compressive stress (tone) in the arterial media that may induce VV deformation and wall hypoxia has been assessed indirectly through pulse wave velocity (PWV) measurements relying on the classical Moens–Korteweg formula that relates PWV to the elastic modulus (Young's modulus *E*) of the wall as described in the below equation, where *h* is the wall thickness, *r* is the artery radius and *ρ* is the density

3.1 $$\text{PWV}=\sqrt{\frac{E\cdot h}{2r\rho }}.$$

Caro *et al*. [29], by means of non-invasive multichannel Doppler ultrasound measurements of PWV, showed that cigarette smoking in healthy subjects increases arterial wall stiffness (*E*). In a related study, Tarnawski *et al*. [30] observed increased PWV in subjects with a nicotine patch. Levenson *et al*. [31] found that PWV was increased significantly in hypertensive subjects compared to normotensive controls. Tarnawski *et al*. [32] determined significantly higher PWV in non-athletes compared to age-matched athletes. Psychosocial stress/anxiety have also been shown to increase PWV [33]. All of these factors that increase PWV are considered risk factors for cardiovascular disease. Szmigielski *et al*. [34] showed that PWV correlates with aortic atherosclerosis as characterized by intimal–medial thickness. The implication of these studies is that increased vessel wall compression associated with elevated PWV (E) compresses VV leading to outer wall hypoxia and vascular dysfunction.

But the link between increased wall stiffness (PWV) and hypoxia does not appear to have been demonstrated directly. There is, however, indirect evidence for such a link. First, PWV as an indicator of arterial stiffness has been validated clinically in large arteries [35]. In addition, with increased pressure in the aorta and increased rigidity in the arterial wall associated with systemic arterial hypertension, VV blood flow decreases, presumably a result of VV compression [18,36] and this results in local hypoxia and aortic medial necrosis. In support of this view, experimental ligation of the VV results in medial aortic necrosis in dogs [37].

### 3.2. The vasa vasorum in atherosclerotic plaque development

The initiation and progression of atheromas [38–40] is characterized by elevated plasma lipid concentration (hypercholesterolaemia) [41] and increased transport of low-density lipoprotein (LDL) across an impaired endothelium [42,43]. Dysfunctional endothelium develops under the influence of hypertension, free radicals and inappropriate flow shear stress. Contrary to the long-standing belief that the vast majority of LDL transport occurs at the luminal side of the vessel, recent studies have shown that adventitial VV play a significant role in the initiation and/or progression of vascular disease [44,45]. Endothelial dysfunction in both the arterial lumen and the arterial VV leads to delivery of LDL, oxidized and inflammatory products, at a rate [46] greater than the clearance by VVV [47]. Phagocytes are attracted into the vessel wall, and a chronic vascular inflammatory environment (i.e. increased expression of NF-κB) initiates the process of local plaque formation [48]. Another consequence of infiltration of lipids, accumulation of macrophages, release of cytokines and angiogenic stimulus generated by oxidative stresses in the vessel wall is the proliferation of VV [49–53]. Accumulation of inflammatory cells in the intima stimulates the proliferation of smooth muscle cells in the media, and as a consequence, the vessel wall undergoes positive remodelling, increasing its wall thickness.

Thickening of the vessel wall changes the transmural pressure gradient and wall tissue stresses, and creates regions with low oxygen tension in the medial layer [54,55], where metabolic needs exceed the amount of oxygen that can diffuse from the lumen [56,57]. The blood flow resistance is high in the distal VV located close to the media layer. High wall tissue stresses within the intima and medial layers of the wall can exceed the blood pressure in VV at that location, and significantly restrict blood flow through them. Also, VV blood flow may be selectively reduced by increased smooth muscle tone in proximal VV due to inflammation or thrombosis. These mechanisms can result in insufficient removal of waste products and local hypoxia in the media layer.

Reduced perfusion from VV promotes increased progression of fatty streaks, further increasing oxygen demand [58] in the intima layer, resulting in local hypoxia. Thus, higher VV density is required to meet the oxygen perfusion needs of the arterial wall. Hypoxia triggers local VV neovascularization via the production of angiogenic factors. Studies in hypertensive rats demonstrated increase in HIF-1α and angiogenic factors [49,51–53], including VEGF expression in the aorta, which was subsequently followed by increase in VV density around the aorta [59]. A similar increase in HIF-1α and VEGF has been demonstrated in coronary arteries in hypercholesterolaemic pigs [60]. The reversibility of endothelial dysfunction at the early stages of atherosclerosis is associated with a reduction in VV density (neovascularization). Moreover, anti-angiogenic therapy reduced plaque neovascularization and plaque growth [61], and it was associated with a reduction of macrophages in the plaque and around the VV [20,62].

Neovascularization within atherosclerotic plaques leads to VV that are immature. These vessels lack a smooth muscle cell layer, tight junctions and a continuous basement membrane [63]. Dysfunctional VV lead to stasis of blood flow, loss of endothelial barrier function, increased vascular permeability and extravasation of fluids and proteins [64,65]. Unfortunately, proliferation of ruptured and/or leaky VV seems to facilitate the ingress of pro-atherogenic cellular and soluble plasma components, including macrophages and inflammatory factors into the vessel wall, thus further enhancing angiogenesis [62] and the progression of atherosclerosis [66]. Leaky VV within the atheroma core lead to intraplaque haemorrhage [63,67,68]. Extravasated erythrocytes come in contact with plaque lipid, and undergo haemolysis followed by oxidation of haemoglobin and release of free haem or iron, which accumulates within the plaque [69]. Also, haemoglobin/haem released from phagocytosed erythrocytes by macrophages contributes to the iron deposition in lesions [70], necrotic core size and increased macrophage density [71]. VV haemorrhage is a key factor in the development of unstable atherosclerotic lesions [71,72]. VV is twofold denser in vulnerable plaques and fourfold denser in ruptured plaques, when compared with stable plaques [73,74].

In summary, initiation and progression of VV neovascularization in atherosclerosis is driven by both chronic inflammatory and hypoxic environments within the tissue [75,76]. Angiogenesis of VV is a physiological response of the organism to restore the appropriate nutrition and oxygen supply in the vessel wall; however, dysfunctional VV neovascularization (i.e. increased intraplaque VV density with impaired endothelium) further contributes to plaque inflammation [77], intraplaque haemorrhage [71], thin-cap fibroatheromas [68] and acute cardiovascular events [63,68].

### 3.3. Transport from the lumen

**Figure 3.**
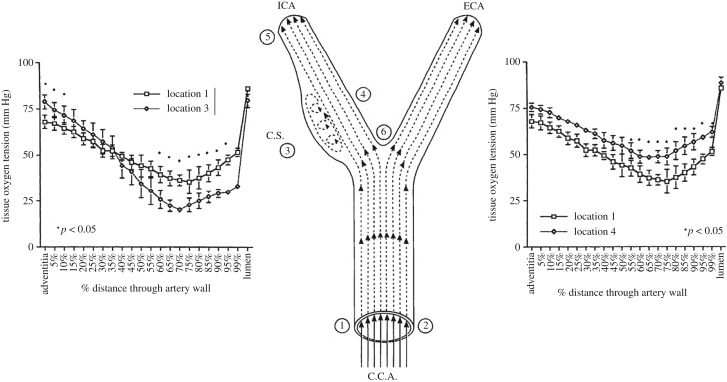
Intra-arterial oxygen tension profiles in dog carotid artery. (Left) Comparison of profiles in the common carotid (position 1) and the disturbed flow carotid sinus (position 3). (Right) Comparison of profiles in the common carotid (position 1) and the stable flow inner wall (position 4). From Santilli *et al*. [17].

Experiments in dogs [17] (figure 3) show significant induction of inner wall hypoxia in the disturbed flow region of the internal carotid artery (carotid sinus—region 3) compared to the common carotid artery (region 1), and reduction of inner wall hypoxia in the stable flow region of the internal carotid (region 4). The minimum *P*O_2_ in region 3 is about 21 mm Hg, region 1 is 38 mm Hg and region 4 is 46 mm Hg. It has been shown that there is a steep rise (fourfold) in HIF-1α concentration in human umbilical vein endothelial cells (HUVECs) as *P*O_2_ levels drop from 38 mm Hg (5%) to 23 mm Hg (3%) [78]. In a recent review paper, 3% oxygen (23 mm Hg) has been described as moderate hypoxia, whereas 5% (38 mm Hg) is considered borderline hypoxic [2]). Thus, it appears that the *P*O_2_ of 21 mm Hg in the carotid sinus represents a hypoxic state relative to the inner wall (46 mm Hg) and the common carotid (38 mm Hg).

**Figure 4.**
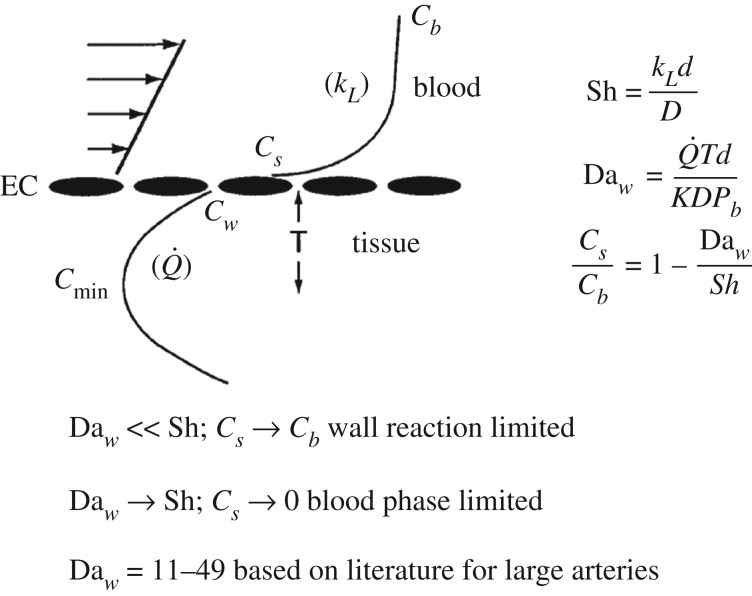
Outline of simplified mass transport considerations for oxygen transport to a blood vessel wall. The schematic displays the concentration profile from the bulk blood (*C_b_*) to the endothelial cell surface (*C_s_*) to the inner wall (*C_w_*) and the minimum value (*C*_min_) as indicated in figure 2. *k*_L_ is the fluid phase mass transport coefficient, $\dot{Q}$ is the oxygen consumption rate and *T* is the distance to *C*_min_—roughly ½ the wall thickness. The velocity profile at the left is linearized because of the thin concentration boundary layer (Leveque approximation) [81].

The hypoxic region in the carotid sinus (region 3 in figure 3) has been shown to be a region of low fluid wall shear stress (WSS) compared to the common carotid artery, whereas region 4 is characterized by higher shear stress [79,80]. These observations suggest that fluid phase transport of oxygen to the blood vessel wall is controlling oxygen tension in the inner wall region. This hypothesis is suggested by the Leveque theory of mass transport in thin boundary layers [81], indicating that the rate of fluid phase oxygen transport to the wall, as characterized by the mass transport coefficient *k_L_* (figure 4), is proportional to the wall shear rate (WSS/viscosity) to the 1/3 power. And, it is well known that region 3 develops atherosclerotic plaques, whereas regions 1 and 4 are typically spared [82–84].

The theory of fluid phase transport to an artery wall has been elaborated in several studies, many in the context of LDL transport. See, for example, representative cases of ‘wall free models’ [85], ‘single layer models’ [86] and ‘multilayer models’ [87]. These studies have also examined the importance of non-Newtonian blood flow modelling on transport and found non-Newtonian effects to be of minor importance. For models of oxygen transport, the early paper of Crawford *et al.* [88], followed by the study of Moore & Ethier [89] and more recently Murphy *et al*. [90] are representative of developments in oxygen transport modelling. Tarbell [91] presented a simplified single layer model that is summarized in figure 4. By assuming that endothelial cells offer no transport resistance for highly diffusible oxygen (*C*_w_ = *C*_s_) and that oxygen consumption by smooth muscle cells in the wall is the limiting rate process within the wall (oxygen diffusion is rapid), a simple expression for the surface concentration relative to the bulk concentration is obtained

3.2 $$\frac{C_{s}}{C_{b}}=1-\;\frac{\text{D}{\text{a}}_{w}}{\text{Sh}}.$$

In the above equation, the Damhkoler number (Da*_w_*) is the dimensionless wall oxygen consumption rate and the Sherwood number (Sh) is the dimensionless fluid phase mass transfer coefficient. The Sh or dimensionless mass transfer coefficient (Sh = *k_L_d*/*D*) introduces the fluid phase mass transfer coefficient (*k_L_*), vessel lumen diameter (*d*) and oxygen diffusion coefficient in the tissue (*D*). The Damkholer number $(D{\text{a}}_{w}=\dot{Q}Td/KDP_{b})$ is the dimensionless oxygen consumption rate ($\dot{Q}$) within the wall: *T* is the half-thickness of the wall and *P_b_* is the bulk oxygen tension (related to *C_b_* through the Henry's law relationship *C* = *KP*, where *K* is the Henry's law constant).

For a fixed oxygen consumption rate (fixed Da*_w_*), when the fluid phase mass transfer rate is large (Sh ≫ Da_*w*_) then *C_s_* → *C_b_* and there is no limitation from fluid phase mass transport (the wall consumption is limiting). However, for lower rates of fluid phase transport, Sh → Da_*w*_, and *C*_*s*_ → 0 (the blood phase mass transport is limiting). Note that equation (3.1) predicts negative surface concentrations when Sh < Da*_w_*. This derives from an assumption in the analysis that *P*O_2_ in the tissue is well above the value of the Michaelis constant in a Michaelis–Menten description of the kinetics of oxygen consumption. The Michaelis constant is typically of order 1 mm Hg [92] and *P*O_2_ in the deep wall is well above that level (figure 2). Values for Da*_w_* based on data in the literature for oxygen consumption in arteries range between 11 and 49 [91].

**Figure 5.**
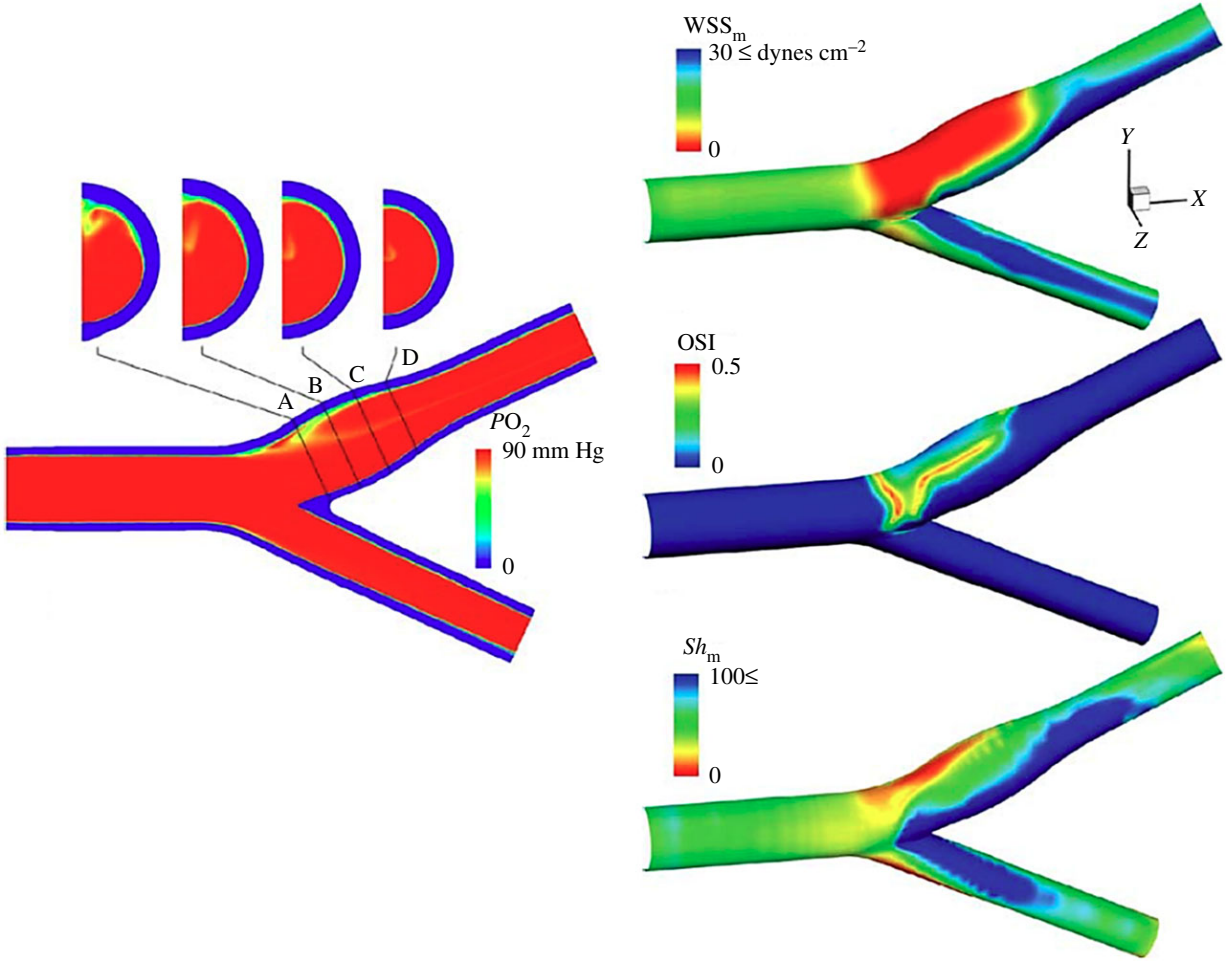
Computer simulations of fluid phase oxygen transport in the carotid bifurcation. Colour coding on the left panels indicates *P*O_2_ levels. Note in the right panel that red is indicative of low Sh and blue of high Sh. Based on Tada & Tarbell [93].

Subsequent detailed computer simulations of oxygen transport in the carotid bifurcation, neglecting oxygen binding to haemoglobin [93], revealed more clearly the fluid phase transport limitations in the carotid sinus (figure 5). The colour-coded concentration profiles show a uniform *P*O_2_ entry profile at 90 mm Hg. The wall boundary condition was taken to be zero, consistent with an assumption of fluid phase transport limitation. The cross-sectional oxygen concentration distributions are shown in the four panels at the top of the figure. There is a clear prediction of reduced fluid phase oxygen concentration in the carotid sinus region that is consistent with the observations in figure 3. The Sh calculations show regions of highly reduced Sh in the carotid sinus along with regions of elevated Sh on the opposite wall—again quite consistent with the data in figure 2. Note also that regions of low Sh co-localize with regions of low WSS, although there is not a complete overlap of the two regions (figure 5 right). Moore & Ethier [89] had pointed out inaccuracies associated with the neglect of haemoglobin binding, but they do not alter the basic conclusions of Tada & Tarbell [93].

Related computer simulations for a curved artery approximating the curvature of a coronary artery over the surface of the heart have been described [94]. For typical coronary artery curvatures, flow conditions and transport properties characteristic of oxygen, the results indicate a large difference in Sh between the outside (Sh about 55) and inside (Sh about 2) walls, implying that O_2_ transport at the inner wall could be limited by the fluid phase. More generally, the inner curvature of arteries is also a region of relatively low WSS [9] and a site for localization of atherosclerosis [95].

## 4. Disturbed flow activates HIF-1α

Although HIF-1α activation by hypoxia is the canonical pathway, interestingly a number of recent studies revealed that HIF-1α can be activated non-canonically, through mechanical activation. Wu *et al*. [11] show that disturbed blood flow (which exerts a low, oscillating shear stress on the surface of endothelial cells lining arteries) in porcine arteries led to elevated levels of HIF-1α. Consistent with this, Feng *et al.* [9] show that HIF-1α was also elevated at sites of disturbed flow in porcine and murine arteries. A study by Fernandez Esmerats *et al*. [96] shows that HIF-1α was enhanced in endothelial cells exposed to disturbed flow located at the fibrosa side of the aortic valve of human patients. These studies revealed that HIF-1α can be activated by disturbed blood flow *in vivo.* In addition, these studies, using *in vitro* flow systems that recapitulate the shear stress *in vivo*, show that endothelial cells exposed to disturbed flow under normoxic conditions had elevated HIF-1α mRNA and protein levels [9,11,96]. Thus, it appears from these findings that both hypoxia induced by disturbed flow, and disturbed flow itself can induce HIF-1α expression.

The activation of HIF-1α by disturbed flow is linked to the induction of endothelial cell dysfunction and subsequent atherogenesis and calcific aortic valve disease. In response to disturbed flow, HIF-1α has been shown to be activated through NFкB proinflammatory signalling, along with stabilization by the debiquitinating enzyme cezanne [9]. Elevated HIF-1α levels in turn trigger the activation of glycolysis genes, leading to a metabolic reprogramming of cells and elevated levels of inflammation and proliferation, and these in turn are hallmarks of a dysfunctional endothelium which promotes atherogenesis [9,11]. Wu *et al.* [11] suggest that the mechanism by which disturbed flow activates HIF-1α is through reactive oxygen species (ROS) generating oxidase NOX4, which leads to elevated HIF-1α levels and downstream activation of proinflammatory NFкB signalling, enhanced glycolysis and endothelial cell dysfunction. Interestingly, HIF-1α activation by disturbed flow can be controlled epigenetically. Fernandez Esmerats *et al.* show that downregulation of miR483 by disturbed flow leads to elevated HIF-1α levels in endothelial cells through enhanced expression of a miR483-target gene UBE2C, which functions as an ubiquitinating protein that degrades VHL, leading to HIF-1α accumulation. Elevated HIF-1α levels promote inflammation and endothelial–mesenchymal transition in endothelial cells, leading to dysfunction and calcification in the aortic valve [96].

Non-canonical activation of HIF-1α by disturbed flow appears to play an important role in the progression of disease. However, it also appears that these pro-atherogenic disturbed flow areas are hypoxic, and that disturbed flow along with hypoxia play a dual role in switching on HIF-1α-mediated endothelial cell dysfunction leading to disease progression in arteries.

### 4.1. Atherosclerosis is not common in veins

If hypoxia plays a role in atherosclerosis, and *P*O_2_ is much lower in venous blood than arterial blood, why is atherosclerosis not common in veins? While this question appears not to have been answered conclusively in the literature, several contributing mechanisms have been described. Even though the *P*O_2_ of venous blood (about 40 mm Hg) is much lower than arterial blood (about 100 mm Hg), vein wall oxygenation is achieved by diffusion of oxygen from blood in the lumen as well as the VV, much like arteries [97]. The VV of saphenous veins and tributaries, for example, originate from feeding arteries in the surrounding adipose tissue and penetrate into the media and adventitia of the vein wall providing arterial levels of oxygen to the outer wall regions. Thus, the outer layers of veins may experience arterial oxygen levels. It has been suggested that defects in this mechanism lead to vein wall hypoxia that is a contributing factor for varicose vein formation [98]. Varicose veins are characterized by intimal hyperplasia and smooth muscle cell dysfunction without the lipid accumulation associated with atherosclerotic plaques in arteries [99].

In the venous system of the lower limbs, a series of bicuspid valves ensure blood flow movement in the cephalad direction, preventing the reflux of blood towards the feet while in the upright posture. The venous valves are characterized by flow separation from their leaflets leading to disturbed flow that tends to be atherogenic in arteries (recall figure 3). Disturbed flow may lead to clotting and deep vein thrombosis but not lipid deposition and atherosclerosis [100].

Veins are generally protected from lipid deposition. In an early work, McCluskey & Wilens [101] observed no trace of lipid in the intima or media of any sections prepared from veins removed from 20 patients. A plausible mechanism for this observation was presented later by Lever & Jay [102]. They determined that the medial layer of the inferior vena cava was about one-third the thickness of the media in the carotid artery of rabbits. In addition, the porosity of the medial layer of the vena cava to macromolecules was about 10 times that of the carotid artery. They suggested that the thin medial layer with high porosity in veins compared to arteries may permit easy drainage of macromolecules (LDL) preventing their accumulation in the wall, thereby contributing to the low susceptibility of these vessels to atherosclerotic disease.

## 5. Stent hypoxia

The most reliable treatment for symptomatic vascular narrowing is percutaneous transluminal angioplasty (PTA), which widens the narrowed sections of the artery using a catheter balloon and often placing a medical stent, a slender, expandable, cylindrical metal mesh in the region of vascular occlusion [103]. The stent works as a mechanical support for the vascular wall, re-opening the pathological region and restoring the original blood flow. However, PTA with stenting is often complicated by in-stent restenosis secondary to neointimal hyperplasia, which may result in failure of the implant. Although restenosis is linked to different aspects of the procedure, recent studies have highlighted the relationship between reduced levels of oxygen tension within the stented artery wall and intimal hyperplasia [90,104–107]. The stent itself plays a key role in determining the overall oxygen supply to the underlying tissue. Even though the stent restores the required blood flow to downstream vasculature, it may induce a hypoxic condition for the arterial layers around the stent.

**Figure 6.**
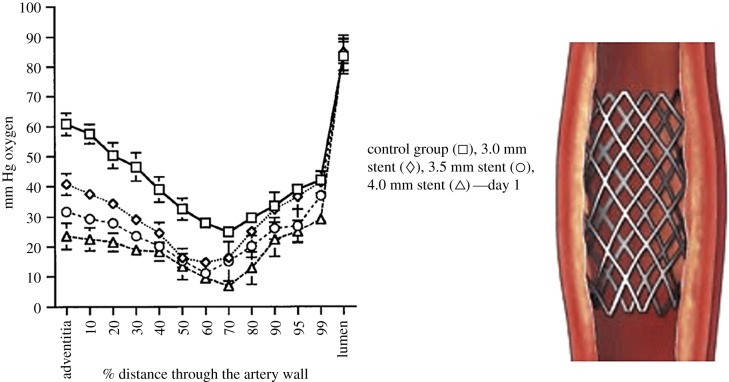
Stent expansion induces outer wall hypoxia. Oxygen tension data at various stent expansions in the distal aorta of rabbits at 1 day after stenting [106].

It is widely believed that intimal hyperplasia following stenting is the result of an inflammatory response to intimal damage during stent implantation and expansion [108,109] as well as a material reaction in the case of polymer-coated stents [110]. The initial IH response occurs rapidly (within one week) and may become significant at one to three months. In addition, decreased oxygen tensions have been noted throughout the artery wall immediately following stent deployment with a return towards control values at 28 days in a rabbit model (figure 6). Larger stent deployment diameters yielded acutely lower artery wall oxygen tensions.

Inner wall hypoxia during stenting appears to be driven by increased smooth muscle cell oxygen consumption associated with cellular compression during the acute phase of response (days) after stent deployment [106]. This increased demand for oxygen can lead to a supply limitation from the blood phase as discussed in the context of figure 2. In addition, inner wall hypoxia may be associated with reduced fluid phase transport driven by local flow separation around stent struts [90,111]. The endothelial layer is also damaged during stenting [112], but the endothelium itself offers very little resistance to oxygen transport and is not expected to be influential in controlling wall hypoxia [91].

Outer wall hypoxia appears to be driven by reduced oxygen transport through the VV. It has been suggested, but not observed directly, that VV deformation associated with increased wall compression in stenting is responsible for outer wall hypoxia. This mechanism would be exaggerated in stent overexpansion as possibly reflected in Santilli *et al*. [106] showing greater outer wall hypoxia with stent overexpansion (figure 6).

Cheema *et al*. [105] showed that outer wall hypoxia after stenting leads to upregulation of VEGF and PDGF inducing an angiogenic response that ultimately increases outer wall microvessel density over a period of four weeks. This may in part account for the observed reduction in outer wall hypoxia after four weeks of stenting observed by Santilli *et al*. [106]. However, this mechanism provides a new pathway for inflammatory response, macrophage infiltration and associated wall thickening that may exacerbate intimal hyperplasia as discussed in the earlier section on VV in atherosclerotic plaque development.

Consistent with evidence for wall hypoxia after stenting is the recent report of Li *et al*. [113] who showed that hyperbaric oxygen therapy after coronary stenting improved patients' myocardial circulation compared to a control group with stents but without oxygen therapy. Earlier work in rabbits had shown that supplemental oxygen reduces IH after stent deployment [107]. Additional work in rabbits indicated that supplemental oxygen inhibits IH and SMC proliferation after creation of an arterio-venous fistula and prolongs patency [114].

### 5.1. Deformation of vasa vasorum after stenting

Previous research has focused its attention on understanding the effect of disruption of blood flow through VV [115–117]. The results are often medial necrosis, stagnant interstitial fluid, decreased vascular wall nutrition and wall hypoxia. All of these may occur when the artery is stent-expanded. Stent-expanded arteries may reach sufficiently elevated degrees of deformation such that high circumferential and radial stresses could compress VV, thus diminishing blood flow through VV and reducing oxygen delivery to the outer layers of the vessel wall. During stent expansion, circumferential stresses in the layers remain rather small as long as the elastic fibres are able to stretch. Once they reach the maximum extension, the stress begins to increase exponentially as collagen, which is 100–1000 times stiffer than elastin, bears increasing load [106]. On the other hand, radial stress is the compressive component responsible for squeezing the structures in the artery wall. Increments in both circumferential and radial stresses likely affect the original VV morphology, and may contribute to artery wall hypoxia. This mechanism naturally implies a relation between final stent diameter, arterial stress and VV diameter that could provide the link between transarterial wall oxygen gradient and the degree of stent expansion [106,117]. In clinical practice, stents are routinely expanded under fluoroscopy to achieve a stent/artery luminal diameter ratio of 1.1 : 1. However, it can happen that the implant exceeds this limit, reaching a situation of overexpansion where the ratio is 1.2 : 1 or greater. Stent expansion may compress the VV, resulting in reduction of vascular wall blood perfusion leading to wall hypoxia. Previous studies have used finite-element analysis (FEA) to investigate the mechanical interaction between expanded stents and atherosclerotic tissues [118–120]. Others have focused on the influence of the implant design on the haemodynamics and oxygen transport rates through the stented lumen [90,117].

**Figure 7.**
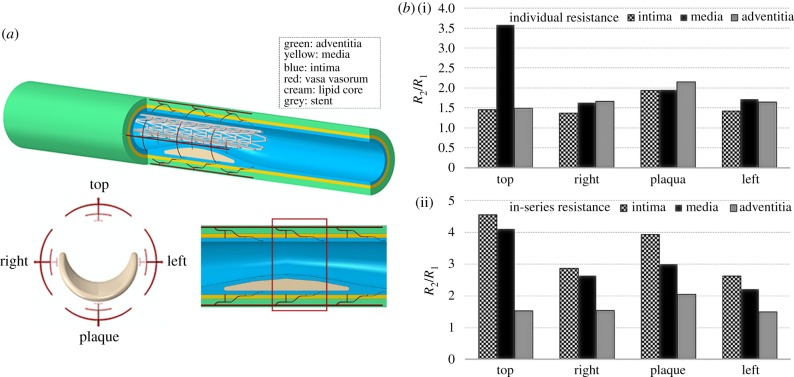
(*a*) Illustration of the plaque, VV, stent model in lateral section. (*b*) Fold increase in resistance to flow (pressure drop/flow) for over-expanded stent (1.3 : 1). Histograms report both the individual change in resistance at each layer (i) and the resulting augmentation due to the vessels in-series layout (ii). Results refer to VV in the central region in the red rectangle (*a*) [121].

A very recent study implemented FEA to explore VV compression induced by stenting, assessing whether the final stent diameter could induce a hypoxic situation in the outer vascular layers [121]. An idealized multi-layered fibroatheroma model was created (figure 7*a*) comprising the intima, media and adventitia layers as thick-walled nonlinear elastic cylindrical tubes. The plaque was modelled as a semi-annular lipid core placed in the intimal layer, causing a 60% stenosis. Four VV trees were oriented symmetrically around the vessel (figure 7*a*) with three branches of decreasing diameter in the axial and radial direction, penetrating into each vascular layer. The stent was expanded to reach a stent/artery lumen diameter ratio of 1.1 : 1, 1.2 : 1 and 1.3 : 1 in different simulations. The results indicated large increases in resistance to blood flow (pressure drop/flow) with stent expansion. Assuming the pressure drop between the VV inlet and outlet remain constant, the blood flow would drop more than four times in the media and intima when considering the in-series network organization of longitudinal, descending branches (figure 7*b*). These increases in hydraulic resistance could potentially lead to a hypoxic condition, especially when the stent is over-expanded.

### 5.2. Altered stent geometry enhances fluid phase mass transport

**Figure 8.**
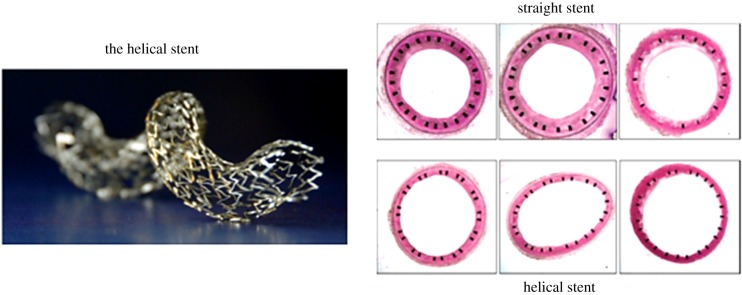
The helical stent reduces intimal hyperplasia. (*a*) Image of a helical stent; (*b*) reduction of intimal hyperplasia 30 days after helical stent implantation in pig common carotid arteries [104].

In addition to anti-proliferative [122] and anti-inflammatory [123] drug release from stents to suppress IH, and hyperbaric oxygen to directly ameliorate the effects of hypoxia on IH after stenting [113], an approach based on alteration of blood flow mechanics and blood phase oxygen transport rates was introduced by Shinke *et al*. [124] and described in greater detail by Caro's group [104,125,126]. They modified a commercially available bare-metal, nitinol, self-expanding stent by introducing a helical-centreline geometry (figure 8*a*). The helical geometry induces secondary flows (in the plane perpendicular to the axial flow) that alter fluid shear stress and mass transport characteristics. The new stent design was initially tested in straight sections of pig common carotid arteries with a conventional straight centreline stent in one CCA and the helical stent in the contralateral CCA. After 30 days, there was a 45% reduction in intimal thickness associated with the helical stent compared to the straight stent (figure 8*b*). In addition, there was a 40% reduction in the number of adventitial microvessels associated with the helical stent, suggesting a reduction in wall hypoxia. A subsequent human clinical trial of patients with peripheral artery disease showed improved patency of the superficial femoral artery out to two years when treated with a helical stent compared to a straight stent [127]. All of this is consistent with recent studies from De Nisco *et al*. [128] suggesting the atheroprotective nature of helical flow in the coronary arteries.

Computer simulations of flow in the helical stent versus the straight stent [125,126] showed that the helical stent enhances WSS and mass transfer rate (indicated by the Sh) significantly—about threefold for both WSS and Sh. In addition, it was shown that shear stress gradients in the peripheral direction are induced by secondary flow in the helical stent, whereas there are no peripheral gradients for straight stents. Increased WSS has been shown to suppress IH [129,130]. It has been demonstrated that increased nitric oxide production, a normal response of endothelial cells to increased WSS, suppresses smooth muscle cell proliferation, reducing oxygen consumption and migration from media to intima [131]. These mechanisms contribute to reduced IH in high WSS. The possible effects of shear stress gradients, independent of shear stress magnitude, on stent performance are not known. Although wall oxygen tensions were not measured, it is expected that elevated mass transfer rates (Sh) reduce wall hypoxia (recall figure 4). Estimation of the level of reduction through modelling has not been attempted, but would require measurements of smooth muscle and fibroblast oxygen consumption rates beneath an expanded stent as critical input information. Then the simple model described in figure 4 or more sophisticated models could be examined to estimate oxygen tensions within the wall.

## 6. Summary

1. Hypoxia in the vascular wall leads to the upregulation of the transcription factor HIF-1α that induces pro-atherogenic genes enhancing cell proliferation, inflammation and angiogenesis.
2. Oxygen is transported to the intima and inner media from luminal blood flow and to the adventitia and outer media by VV blood flow.
3. Elevated compressive stresses in the outer layers of the wall associated with hypertension, smoking and other cardiovascular risk factors reduce blood flow by compressing the VV.
4. Adventitial VV play a significant role in the initiation and progression of vascular disease.
5. Dysfunctional VV neovascularization contributes to plaque inflammation, intraplaque haemorrhage, thin-cap fibroatheromas and acute cardiovascular events.
6. Disturbed blood flow in vascular bifurcations and curvatures leads to reduced oxygen transport from blood to the inner layers of the wall. These regions of disturbed flow are associated with the development of atherosclerotic plaques.
7. HIF-1α is also activated in disturbed flow by a mechanism that is independent of hypoxia.
8 Both hypoxia and disturbed flow in regions of vessel bifurcation and curvature conspire to upregulate HIF-1α that induces vascular dysfunction and atherogenesis.
9. Vascular stenting to open a diseased blood vessel induces both outer and inner wall hypoxia which are exaggerated by stent overexpansion. This hypoxia leads to neointimal hyperplasia that may evolve to restenosis.
10. Inner wall hypoxia is primarily driven by the increased oxygen consumption of compressed cells (smooth muscle and fibroblasts), although disturbed flow around stent struts may reduce oxygen transport from the lumen in the early response (until the struts are covered by neo-intima). Outer wall hypoxia is secondary to VV compression reducing blood flow to the outer layers in addition to cell compression.
11. Stent overexpansion exacerbates hypoxia by increasing wall stresses and VV compression.
12. To overcome oxygen transport limitations from lumenal blood flow, helical stents have been developed that induce secondary flows to enhance oxygen transport rates. Early animal and human studies of the helical stent show reduced intimal hyperplasia compared to straight stents.

## Data accessibility

This article does not contain any additional data.
